# Single-cell transcriptomics reveals a novel mechanism of RDH16 regulating immune infiltration in hepatocellular carcinoma

**DOI:** 10.3389/fimmu.2025.1689987

**Published:** 2026-01-12

**Authors:** Zhenzhen Zhang, Rui Fan, Jing Ma, Dehui Li, Yan Jiang, Fahui Liu, Qiming Gong

**Affiliations:** 1 Department of Pathology, The Second Affiliated Hospital of Fujian Medical University, Quanzhou, China; 2 Department of Pathology, Hangzhou Linping Hospital of Traditional Chinese Medicine, Hangzhou, Zhejiang, China; 3 Department of Nephrology, Affiliated Hospital of Youjiang Medical University for Nationalities, Baise, China; 4 Key Laboratory of Medical Research Basic Guarantee for Immune-Related Diseases Research of Guangxi, Baise, China; 5 School of Medicine, Xiamen University, Xiamen, China

**Keywords:** hepatocellular carcinoma, single-cell transcriptomics, RDH16, immuneinfiltration, tumor microenvironment

## Abstract

**Introduction:**

Hepatocellular carcinoma (HCC) exhibits pronounced intratumoral heterogeneity and a complex immune microenvironment, which together limit therapeutic efficacy. Identifying actionable biomarkers and mechanisms of immune modulation remains critical for improving patient outcomes.

**Method:**

We integrated single-cell RNA sequencing data with bulk transcriptomic datasets to comprehensively characterize tumor cell heterogeneity and immune landscape features in HCC. Associations between RDH16 expression and clinicopathological characteristics were evaluated, and in vitro functional assays were conducted. Mendelian randomization analyses were performed to assess causal relationships.

**Results:**

RDH16 expression was significantly reduced in HCC tissues compared with non-tumor liver tissues and was associated with vascular invasion, elevated alpha-fetoprotein levels, poor tumor differentiation, and advanced T stage. Higher RDH16 expression was consistently associated with improved overall prognosis. Functional assays indicated that RDH16 did not directly affect tumor cell proliferation, migration, or invasion. Notably, RDH16 expression was inversely correlated with infiltration of CD163⁺ M2 macrophages, suggesting a potential immunomodulatory role in the tumor microenvironment. Mendelian randomization analyses further supported a protective effect of higher RDH16 expression against HCC risk.

**Discussion:**

These findings identify RDH16 as an immune-modulating biomarker in HCC, highlighting its potential role in shaping the tumor immune microenvironment and suggesting new avenues for personalized immunotherapeutic strategies.

## Introduction

Hepatocellular carcinoma (HCC) ranks as the second leading cause of cancer-related mortality worldwide, characterized by a high incidence rate and a poor prognosis (1). Tumor heterogeneity remains a critical barrier to immunotherapy. HCC exhibits diversity in genomic and epigenetic changes among cancer cells, accompanied by varying immune cell infiltration in the tumor microenvironment (2, 3). Therefore, deciphering the molecular heterogeneity and immune landscape of HCC is essential for developing precision therapies.

The immune microenvironment in HCC plays a crucial role in tumorigenesis. It is correlated with patient prognosis, characterized by the infiltration of inflammatory cells, including T cells, macrophages, and dendritic cells, among others (4). T cells play a central role in the HCC immune microenvironment, with distinct subsets, such as cytotoxic T lymphocytes (CTLs) and regulatory T cells (Tregs), exerting antagonistic effects on tumor immune evasion and progression. CTLs generally exhibit antitumor effects, while Tregs promote immune evasion (5). Additionally, macrophages display varying functional states in HCC, typically classified as pro-inflammatory M1 and anti-inflammatory M2 types, with the latter being more prevalent in the tumor microenvironment and associated with immune suppression and tumor progression (6, 7).

Single-cell transcriptomics enables the identification of cell subgroups, individual tumor cells, their stemness states, and metastatic characteristics (8, 9). For instance, the integrated analysis of single-cell RNA sequencing and spatial transcriptomics in HCC research has identified three distinct tumor cell subtypes: metabolic, proliferative, and pro-metastatic (10). Single−cell transcriptomics has delineated the heterogeneity and temporal dynamics of breast cancer stem cells during tumor progression. Evidence implicates epithelial lineages with low estrogen-receptor expression as putative cells of origin, and identifies a stem-like epithelial cluster comprising discrete subclones that captures the heterogeneity of this compartment (11). The integration of single-cell and spatial transcriptomics enables high-resolution mapping of cell-to-cell communication within the tumor microenvironment. In colorectal cancer (CRC), ligand-receptor signaling axes involving C5a receptor 1 and its ligand RPS19 emerge as key mediators of stromal–tumor crosstalk, providing new mechanistic insights into CRC progression (12). Similarly, multiomic single-cell and spatial analyses in PDAC have identified COA6 as an OXPHOS-related driver that shapes the immunosuppressive microenvironment and promotes tumor aggressiveness and chemoresistance (13). In gastric cancer, integrative single-cell and spatial profiling has defined platinum-resistant molecular subtypes and highlighted KLF9 as a key regulator whose modulation enhances therapeutic response (14).

RDH16 is a crucial member of the retinol dehydrogenase (RDH) family, regulating the retinoic acid (RA) signaling pathway. RA, a bioactive molecule derived from vitamin A, modulates gene expression by binding to nuclear receptor transcription factors, thereby influencing cell proliferation and differentiation during the progression of HCC (15, 16). Previous studies suggest that RDH16 may regulate tumor−cell proliferation and differentiation through modulation of retinoic acid (RA) signaling. Moreover, RA signaling promotes the differentiation of regulatory T cells, modulates the suppressive activity of myeloid−derived suppressor cells, and drives monocyte−to−macrophage differentiation, thereby shaping the immune landscape of the tumor microenvironment (17–19). These results suggest that RDH16 might influence tumor immune monitoring and evasion by modifying RA concentrations. However, the specific mechanisms underlying RDH16 function in HCC remain unclear. Therefore, elucidating the biological roles and molecular mechanisms of RDH16 in HCC will help clarify the contribution of RDH16-mediated RA signaling to tumorigenesis and progression and may provide new potential therapeutic targets for HCC treatment.

This study aims to employ multi-omics approaches to investigate the heterogeneity of tumor cells and the characteristics of immune infiltration in HCC. Concurrently, it examines the function of RDH16 within tumor cells and its association with the immune microenvironment of HCC, with the objective of identifying novel molecular markers for HCC treatment.

## Materials and methods

### Acquisition and processing of HCC bulk data

Data on expression profiles and clinical information for liver hepatocellular carcinoma (LIHC) were sourced from the TCGA database. Data for HCCDB18, including expression profiles and clinical information, were retrieved from the HCCDB database (20). The Gene Expression Omnibus (GEO) database (https://www.ncbi.nlm.nih.gov/geo/) was also used to download gene expression data from GSE14520 and other relevant datasets (21). The probes in the datasets were mapped to gene symbols using annotation files. The expression value was averaged if multiple probes were linked to the same gene symbol. In cases where a single probe mapped to multiple gene symbols, the expression data for that probe were excluded. For microarray datasets, only HCC tumor samples with documented survival time and survival status were retained for further analysis.

We utilized the BEST platform (22) (https://rookieutopia.com/) to investigate the correlation between the mRNA expression levels of CCL16 and immune-related genes in tumor samples from multiple HCC cohorts. Additionally, we conducted spatial transcriptome analysis of LIHC using the Sparkle database (https://grswsci.top/). The SpatialFeaturePlot function in the Seurat package was employed to visualize the main cell types in the spatial transcriptome and display the expression information of the RDH16 gene in the spatial transcriptome data. Spearman correlation analysis was used to calculate the correlation between the cell composition of all spots and the expression level of RDH16, and the linkET software package was utilized for visualization.

### Analysis and processing of single-cell RNA sequencing data

ScRNA-seq data comprising 21 samples were obtained from the GSE149614 dataset from the GEO database (23). Among these, 18 liver cancer and adjacent normal samples were retained for analysis. Version 5.1.0 of the Seurat R package was utilized for data processing. The PercentageFeatureSet function calculated the percentage of mitochondrial gene expression for each cell, which was then filtered based on specific criteria: the number of expressed genes was greater than 250, the mitochondrial content was less than 10%, and each cell had a minimum of 500 unique molecular identifiers. Cell type identification was performed using specific markers from previous studies (24) and the CellMarker 2.0 database. Malignant cells were identified by KRT8 and KRT18; fibroblasts by COL3A1, THY1, COL1A2, and DCN; macrophages by SPP1, CD68, and IL1B; T cells by CD3D, CD3E, CD3G, and CD2; Natural Killer (NK) cells by KLRF1, NKG7, and GNLY; Natural Killer T (NKT) cells by KLRF1, NKG7, GNLY, CD3D, CD3E, CD3G, and CD2; B cells by CD79A, MS4A1, CD19, and IGHG1; endothelial cells by PECAM1, VWF, CDH5, and PLVAP; and mast cells by CPA3 and GATA2.

### Molecular subtype identification and heterogeneity analysis

Molecular subtyping of the TCGA-LIHC, HCCDB18, and GSE14520 was performed using the ConsensusClusterPlus R package (parameters: clusterAlg = “pam”, distance = “euclidean”, reps = 500). When K = 2, the samples were divided into two groups designated as C1 and C2. At K = 2, the consensus matrix heatmap exhibited clear and sharp boundaries, indicating stable and robust clustering of the samples. To analyze somatic mutations (SNVs) and clinical characteristics in different subtypes, we used the maftools (v2.4.05) package for mutation data analysis and visualization between risk groups. Genes with a mutation frequency greater than 3% in colon cancer were selected, and oncoPrint from the ComplexHeatmap (25) package was employed for visualization. The distribution of mutations across different subtypes was assessed using the chi-square test. We used five methods to evaluate the immune cell infiltration levels in different subtypes: CIBERSORT, MCPcounter, EPIC, TIMER, and quantise. These methods were applied to assess the immune microenvironment in the TCGA-LIHC dataset and to compare whether there were significant differences in immune infiltration levels across the subtype groups.

### *In vitro* functional validation

The IncuCyte Zoom live-cell imaging system (Essen BioScience, Ann Arbor, MI, USA) was used for proliferation and wound healing assays. HepG2 cells were seeded in 6-well plates and maintained at 37°C in an incubator. Images were captured every 12 hours using a 10× objective with high-definition phase-contrast and fluorescence microscopy. Image analysis and cell quantification were performed using the IncuCyte software. The ability of cell clone formation was evaluated using a colony formation assay. The invasive potential of HepG2 cells was assessed using an *in vitro* invasion assay with a transwell chamber.

### Multiplex immunofluorescence staining

Multiplex immunofluorescence staining of formalin-fixed, paraffin-embedded (FFPE) human HCC and adjacent non-tumoral liver tissue was performed on 4-µm sections. After overnight drying at 60°C to prevent detachment, sections were deparaffinized in fresh xylene (2–10 min) and rehydrated through a graded ethanol series (absolute, 2–5 min; 95%, 3 min; 85%, 5 min; 70%, 5 min), followed by distilled water. Endogenous peroxidase was quenched with 3% H2O2 (10 min, RT), and the slides were washed in PBS (2–3 min). Heat-induced epitope retrieval was carried out in a pH 9.0 Tris-EDTA buffer using a pressure cooker (20 min at pressure, cooled to RT). After serum blocking (10 min), sections were sequentially incubated with primary antibodies for 60 min at RT: CD3 (clone A0452, rabbit polyclonal, Dako, 1:600), CD163 (ab87099, rabbit polyclonal, Abcam, 1:100) and RDH16 (ab224163, rabbit polyclonal, Abcam, 1:1000). HRP-conjugated anti-rabbit secondary antibody (20 min, RT) was followed by tyramide signal amplification using Opal/TSA fluorophores (TSA-N-247259, Akoya Biosciences): PPD650 (pseudo-red) for CD3, PPD620 (pseudo-orange) for CD163, and PPD520 (pseudo-green) for RDH16. Between each round, antigen retrieval was repeated to strip preceding complexes. Nuclei were counterstained with DAPI (15 min, RT), and slides were mounted in enhanced anti-fade medium. Whole-slide images were acquired with a KF-PRO-005-EX scanner (KFBIO) and processed using K-Viewer software.

### Immunohistochemistry

Formalin-fixed, paraffin-embedded 4 µm sections of human hepatocellular carcinoma and adjacent non-tumoral liver were deparaffinised, rehydrated, and subjected to heat-induced antigen retrieval in pH 9.0 Tris-EDTA. Endogenous peroxidase was blocked with 3% H_2_O_2_ and non-specific binding with 5% goat serum (30 min, 37°C). Primary antibodies-RDH16 (ab224163, rabbit polyclonal, Abcam, 1:1000), CD3 (A0452, rabbit polyclonal, Dako, 1:100), and CD163 (ab87099, rabbit polyclonal, Abcam, 1:100) were applied overnight at 4°C. Sections were incubated with HRP-conjugated goat anti-rabbit IgG (Boster SV00002, 1:200, 30 min, RT) and developed with DAB. Counterstaining was performed with haematoxylin before mounting with neutral balsam. For each case, three regions were pre-selected under low power: (i) normal liver, (ii) tumour with high RDH16 expression, and (iii) tumour with low RDH16 expression. CD3^+^ and CD163^+^ cells were counted within each region in five consecutive high-power fields (HPF, 400×) using an Olympus BX43 microscope. Results are expressed as mean ± SD cells/HPF.

### Statistical analysis

Data are shown as mean ± SD unless stated otherwise. Two-tailed unpaired Student’s t-test or Wilcoxon rank-sum test was used for comparing two groups, based on data distribution, while one-way ANOVA was used for analyses with more than two groups. All statistical tests were performed using R (v 4.3.2), with P < 0.05 considered statistically significant.

## Results

### Single-cell atlas of hepatocellular carcinoma

**Figure 1:**
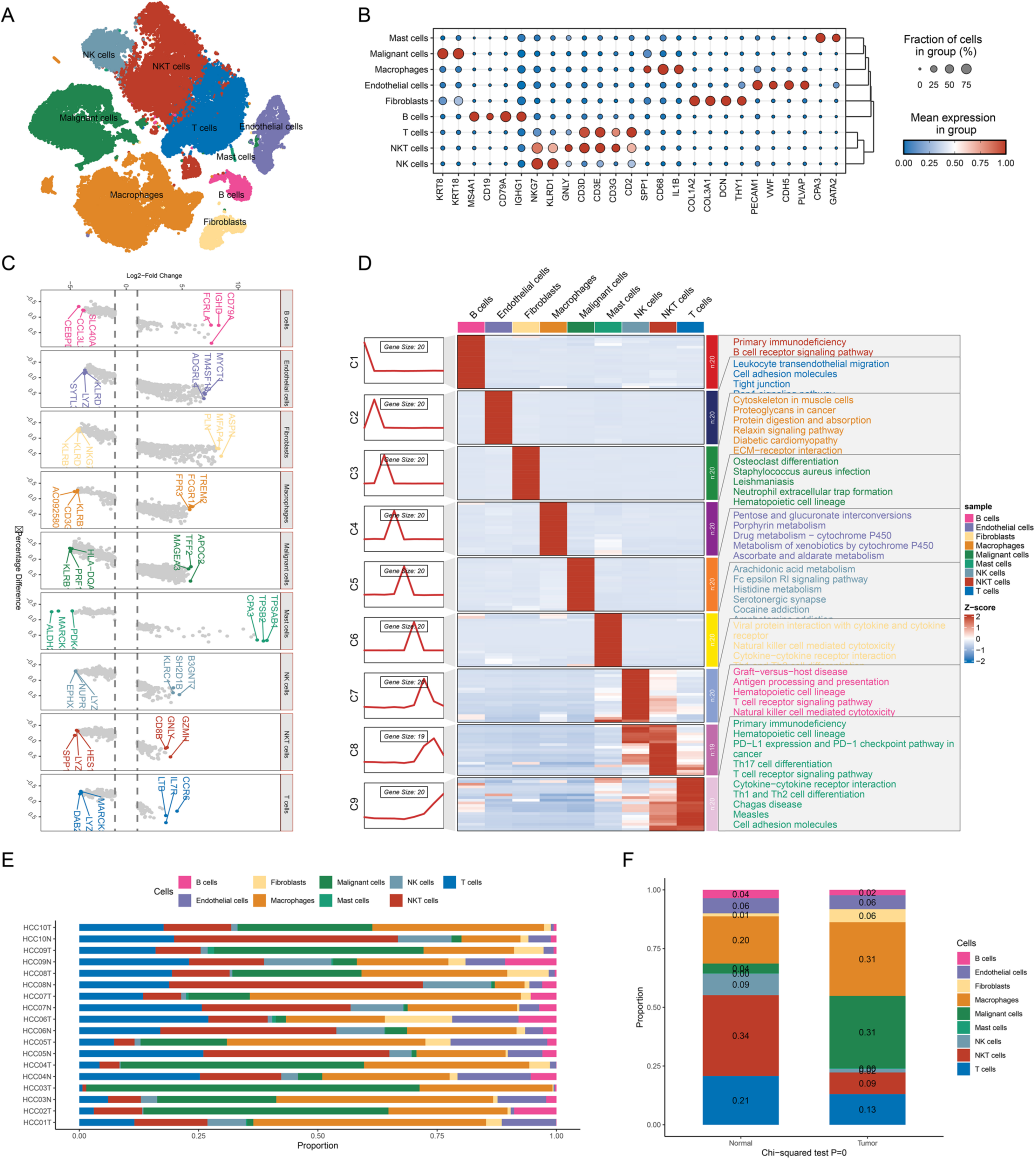
Single-cell transcriptomic profiling delineates the cellular composition of the tumor microenvironment in HCC **(A)** UMAP plot shows main cell types identified by scRNA-seq; **(B)** Dot plot shows the expression of marker genes across cell types; **(C)**.Top 3 marker genes expression analysis for each cell type using the Wilcoxon rank-sum test; **(D)** Pathway enrichment analysis of marker genes across different cell types using the ClusterGVis; **(E)** Proportion of each cell type across individual samples; **(F)** Comparison of cell type compositions between tumor and normal tissues. Statistical significance was assessed using a chi-squared test.

Through single-cell transcriptomic analysis, we clustered cells from HCC samples and annotated cell types based on specific marker genes. The quality control results for the single-cell sequencing data are presented in **Supplementary Figures 1** and **Supplementary Figures 2**. Nine distinct major cell types were identified: NKT cells, NK cells, T cells, B cells, macrophages, fibroblasts, endothelial cells, mast cells, and malignant cells (**Figure 1A**). Differentially expressed marker gene sets for each cell type were further analyzed (**Figures 1B, C**). For instance, T cells expressed high levels of CD3D and CD8A, macrophages expressed high levels of CD68 and CD163, and fibroblasts expressed COL1A1 and ACTA2 at significant levels, confirming the accuracy of our annotations.

Subsequently, we performed functional enrichment analysis for the nine major cell types based on their respective marker genes. The functional enrichment results demonstrated high specificity for each cell type, corresponding to their known biological functions, thereby reinforcing the reliability of our cell type annotations. Specifically, the functional enrichment of B cell marker genes indicated involvement in B cell receptor signaling pathways; fibroblast marker genes were enriched in extracellular matrix remodeling and collagen synthesis; intriguingly, the analysis of malignant cell marker genes revealed that malignant cells in HCC primarily participate in metabolic processes, highlighting the critical role of metabolic alterations in HCC pathogenesis (**Figures 1D**). Further comparative analysis of cell composition between tumor and non-tumor samples (**Figures 1E, F**) revealed significant differences. Notably, tumor cells exhibited a marked increase in tumor samples. Additionally, macrophages and fibroblasts were significantly enriched in tumor samples (P < 0.05), whereas B cells and T cells were more abundant in non-tumor samples (P < 0.05).

Our results provide an initial single-cell landscape of the HCC microenvironment, revealing the enrichment of specific cell types within this environment. Moreover, distinct functional characteristics were observed among different cell types, underscoring the cellular heterogeneity within the HCC microenvironment.

### Subclassification and heterogeneity of malignant cells

**Figure 2:**
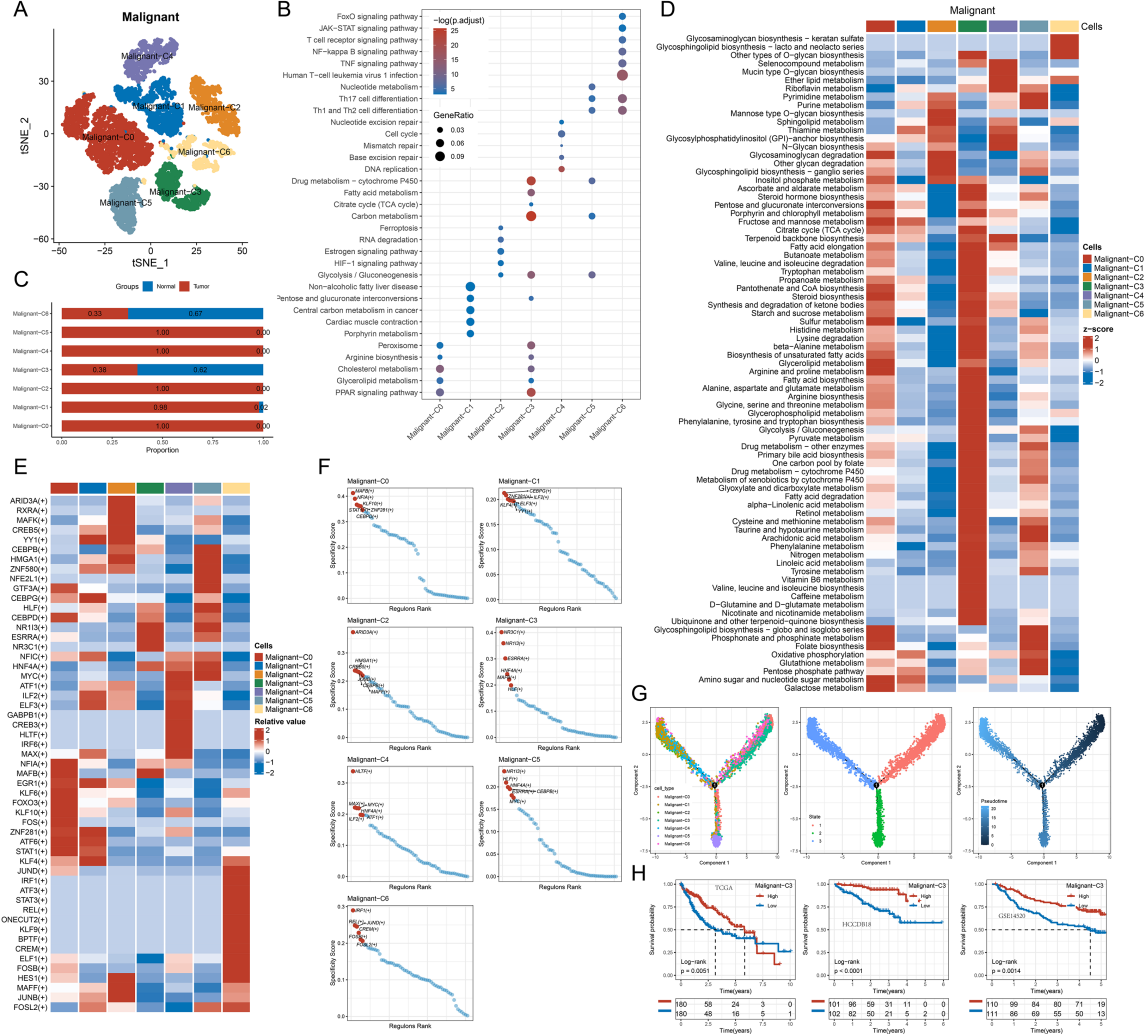
Transcriptional heterogeneity and regulatory characteristics of malignant cell subpopulations. **(A)** t-SNE projection of malignant cells to define intratumoral subclusters based on scRNA sequencing data; **(B)** KEGG pathway enrichment analysis of marker genes in each malignant cell subcluster using clusterProfiler; **(C)** Relative abundance of each malignant subcluster in tumor and normal samples based on single-cell annotations **(D)** Heatmap of metabolic pathway activities in malignant subclusters calculated using GSVA method; **(E)** Heatmap showing the relative activity of key transcription factors across malignant subclusters derived from SCENIC analysis; **(F)** Regulator ranking plots for each malignant subcluster using SCENIC analysis to identify top regulons driving transcriptional programs; **(G)** Monocle 2 analysis of malignant cell subtypes.; **(H)** Survival analysis of patients categorized by gene signatures specific to C3-malignant subclusters based on TCGA cohort data.

Tumor heterogeneity was evident in the initial analysis of the nine cell types. To further explore the heterogeneity of malignant cells, we reclustered them and identified seven subtypes (Malignant-C0 to Malignant-C6; **Figure 2A**). We then performed KEGG pathway enrichment analysis using specific marker genes.

Malignant-C0 was involved in PPAR signaling, lipid metabolism, cholesterol metabolism, arginine biosynthesis, and peroxisomes. Malignant-C1 was enriched in porphyrin metabolism, cardiac muscle contraction, central carbon metabolism in cancer, pentose and glucuronate interconversions, and non-alcoholic fatty liver disease. Malignant-C2 was associated with glycolysis/gluconeogenesis, HIF-1 signaling, estrogen signaling, RNA degradation, and ferroptosis. Malignant-C3 was involved in carbon metabolism, the TCA cycle, fatty acid metabolism, cholesterol metabolism, and drug metabolism - cytochrome P450. Malignant-C4 was enriched in DNA replication, base excision repair, mismatch repair, cell cycle, and nucleotide excision repair. Malignant-C5 was involved in T cell differentiation, carbon metabolism, nucleotide metabolism, and drug metabolism - cytochrome P450. Malignant-C6 was related to human T-cell leukemia virus 1 infection, TNF signaling, and NF-κB signaling (**Figure 2B**). The analysis of the proportion indicates that Malignant-C0, C2, C4, and C5 were mainly found in tumor samples, while Malignant-C1, C3, and C6 were distributed in both tumor and adjacent normal tissues (**Figure 2C**).

To explore metabolic differences, we utilized scMetabolism to analyze single-cell metabolic pathway variations (**Figure 2D**), revealing distinct metabolic profiles for each subtype, which suggests that metabolic reprogramming drives functional differentiation.

SCENIC analysis showed significant differences in the activity of transcription factors (TFs) among the subtypes (**Figure 2E**). Focusing on the top six related TFs for each subtype, their activity profiles revealed the basis of the regulatory network (**Figure 2F**).

Pseudo-time trajectory analysis revealed the development process of malignant cells, identifying three states: Malignant-C3 and C6 were the initial states (state 1), and Malignant-C1 was the terminal state (state 3, **Figure 2G**). ssGSEA scores on the TCGA-LIHC, HCCDB18, and GSE14520 datasets showed that only Malignant-C3 had a significantly different survival curve, with higher scores associated with better prognosis (**Figure 2H**).

### C3 signature correlates with immune regulation and predicts immunotherapy response

**Figure 3:**
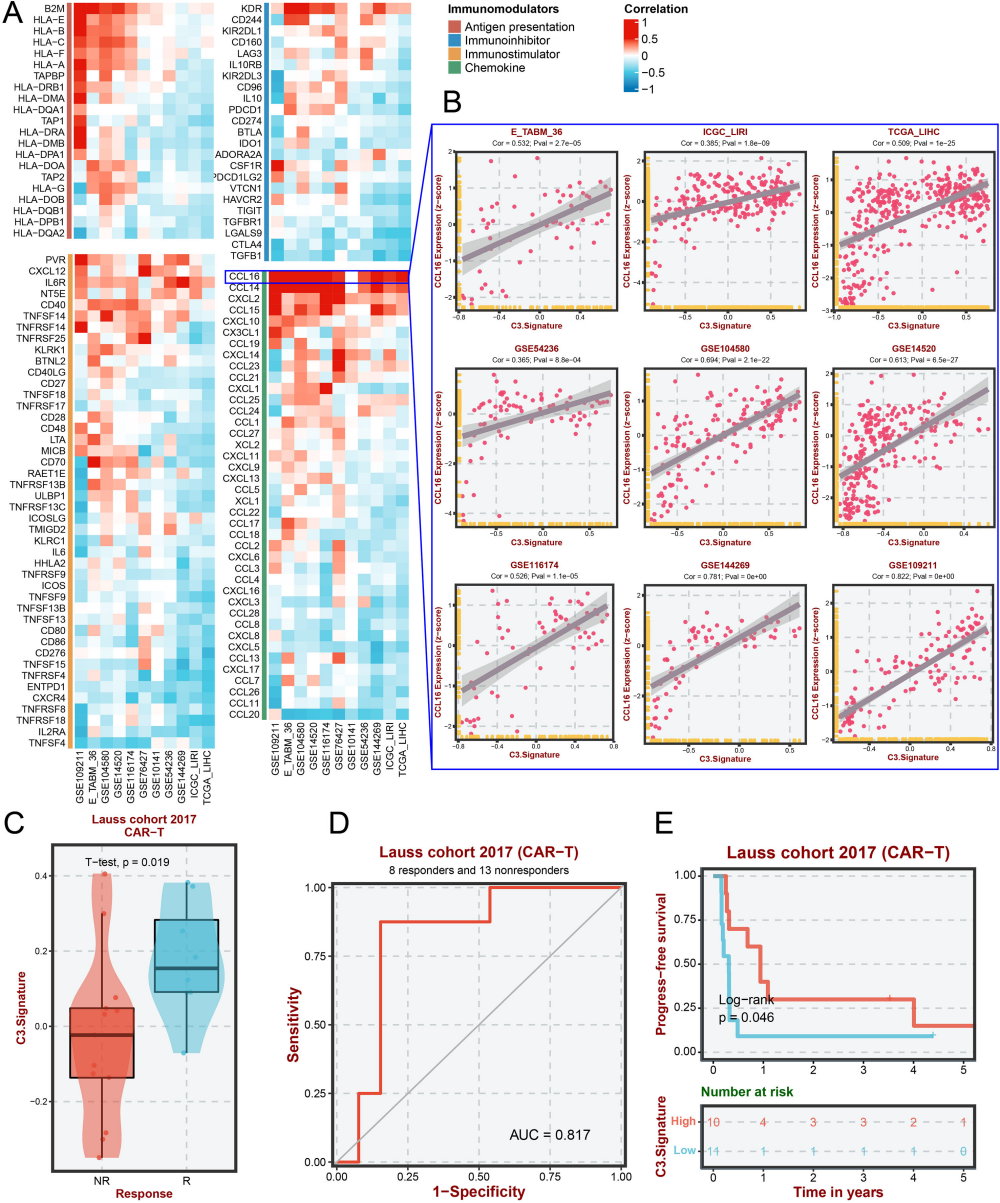
Immunological relevance and clinical predictive value of the C3-malignant. **(A)** Correlation heatmap of C3 signature scores with the expression of immunomodulatory genes across multiple public cohorts, using Spearman correlation analysis; **(B)** Positive correlation between C3 signature scores and CCL16 expression in nine independent HCC cohorts, assessed by Spearman correlation. **(C)** Violin plots comparing C3 signature scores between CAR-T therapy responders and non-responders in the Lauss 2017 cohort, evaluated using a two-tailed t-test; **(D)** Receiver operating characteristic (ROC) curve showing the predictive performance of the C3 signature for CAR-T response in the Lauss 2017 cohort, with an area under the curve (AUC) of 0.817; **(E)**: Kaplan–Meier analysis of progression-free survival in the Lauss 2017 CAR-T cohort, stratified by high and low C3 signature groups. Statistical significance was determined using the log-rank test.

To further explore the biological significance of Malignant-C3, we first assessed the correlation between Malignant-C3 ssGSEA scores and the mRNA expression levels of immune regulatory genes across multiple HCC cohorts. Heatmap analysis revealed that Malignant-C3 scores were significantly associated with various immune modulators, including CTLA4, TGFB1, and CCL20, across multiple HCC datasets (**Figure 3A**). Notably, the Malignant-C3 score showed a strong positive correlation with the mRNA expression of the chemokine CCL16. This association was consistently validated in nine independent public datasets (**Figure 3B**). These findings suggest that the infiltration level of the C3 malignant cell subtype in the tumor microenvironment may be linked to the regulation of immune cell recruitment.

Furthermore, in the Lauss 2017 CAR-T immunotherapy cohort, Malignant-C3 scores were significantly higher in non-responders compared to responders (p = 0.019) (**Figure 3C**). ROC curve analysis demonstrated that the Malignant-C3 score had good predictive power for immunotherapy response, with an AUC of 0.817 (**Figure 3D**). Survival analysis further indicated that patients with high C3 signature expression had significantly shorter progression-free survival (PFS) compared to those with low expression (Log-rank p = 0.046) (**Figure 3E**). Collectively, these results suggest that the Malignant-C3 score reflects an immune-related feature and may serve as a potential biomarker for predicting immunotherapy response and patient prognosis.

### Subtype analysis based on malignant-C3 cells

Prior single-cell analyses identified seven distinct malignant cell subtypes, with the Malignant-C3 subtype exhibiting the most promising potential for further research. Notably, the ssGSEA score of Malignant-C3 was significantly associated with patient survival in multiple transcriptomic datasets, including TCGA-LIHC, HCCDB18, and GSE14520. Therefore, we performed sample clustering analysis using the marker genes of Malignant-C3.

**Figure 4:**
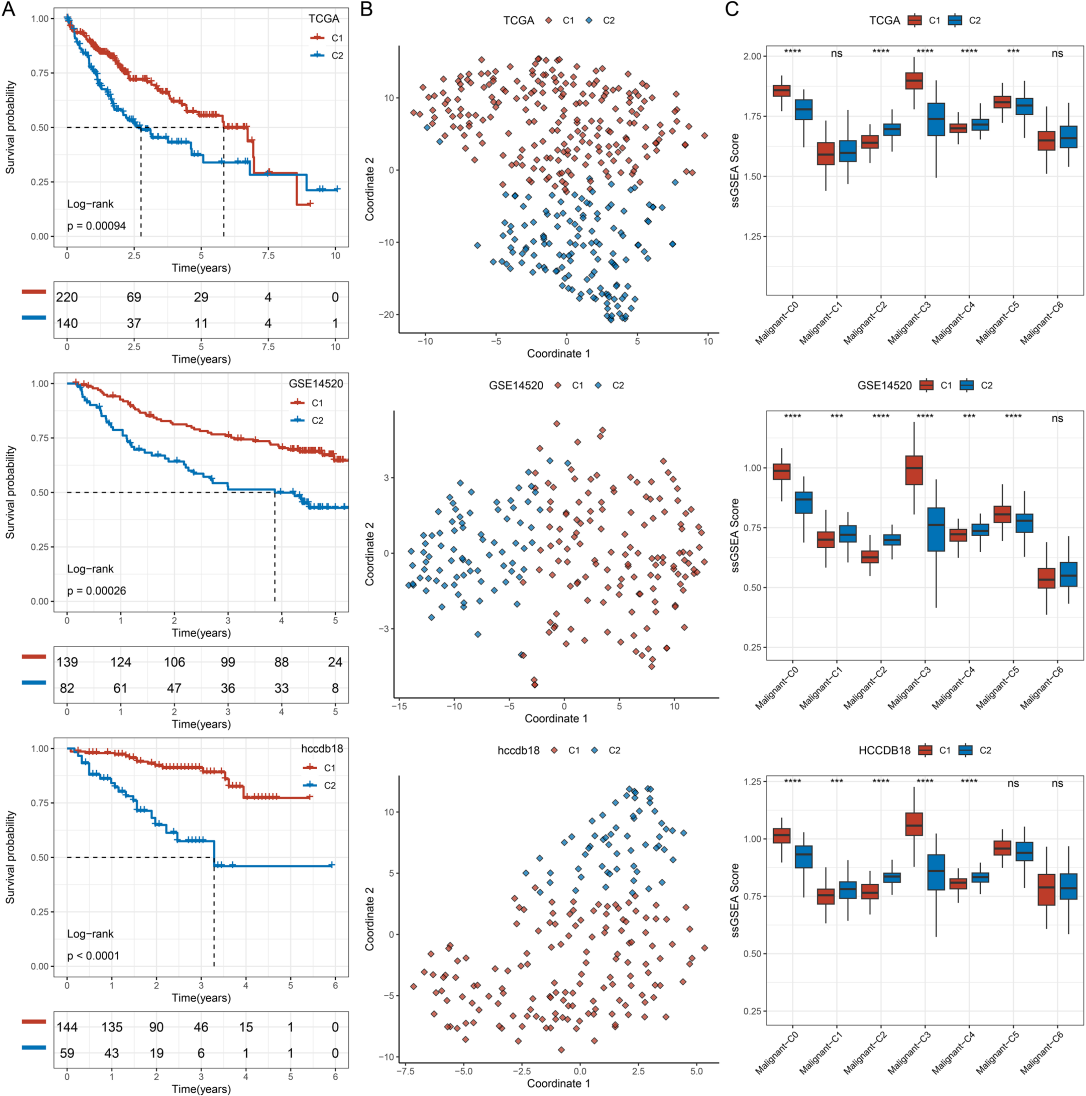
Prognostic significance and malignant subset characteristics of transcriptional subtypes across independent cohorts**(A)** Survival analyses of patients in the GSE14520, HCCDB18, and TCGA-LIHC cohorts stratified by C1 and C2; **(B)** t-SNE of tumor transcriptomes from the TCGA-LIHC, GSE14520, and HCCDB18 cohorts, showing sample distribution according to subtype classification; **(C)** Single-sample gene set enrichment analysis (ssGSEA) comparing the activity of malignant cell subset signatures (Malignant-C1 to C5) between subtypes C1 and C2 across TCGA-LIHC, GSE14520, and HCCDB18 cohorts. ns, p≥0.05; ***p<0.001; ****p <0.0001.

We first conducted univariate Cox regression analysis for the Malignant-C3 marker genes in TCGA-LIHC, HCCDB18, and GSE14520 cohorts, filtering genes with p < 0.05. The intersection of significant genes across the three datasets yielded a set of 39 genes. Then, 39 genes were used to perform consensus clustering for three cohorts, which classified the samples into subtypes. Survival analysis revealed that the two identified subtypes demonstrated significant survival differences across all three cohorts, with the C2 subtype consistently associated with a worse prognosis (**Figure 4A**). To further assess subtype heterogeneity, we applied t-SNE dimensionality reduction to visualize the sample distribution in the GSE14520, HCCDB18, and TCGA-LIHC cohorts. The clear separation between C1 and C2 subtypes in each dataset supports the robustness and reproducibility of our clustering results (**Figure 4B**). In addition, we compared the ssGSEA scores of the seven malignant subtypes between the two clusters. The results revealed that (1): the scoring patterns of all seven subtypes were consistent across datasets; (2) notably, Malignant-C3 scores were significantly lower in the C2 compared to the C1 subtype (**Figure 4C**).

### Genomic and clinical characteristics of C1 and C2 subtypes in the TCGA-LIHC cohort

To further investigate the biological relevance of the C1 and C2 subtypes identified based on Malignant-C3-related gene signatures, genomic alterations and clinical features were compared in the TCGA-LIHC dataset.

**Figure 5:**
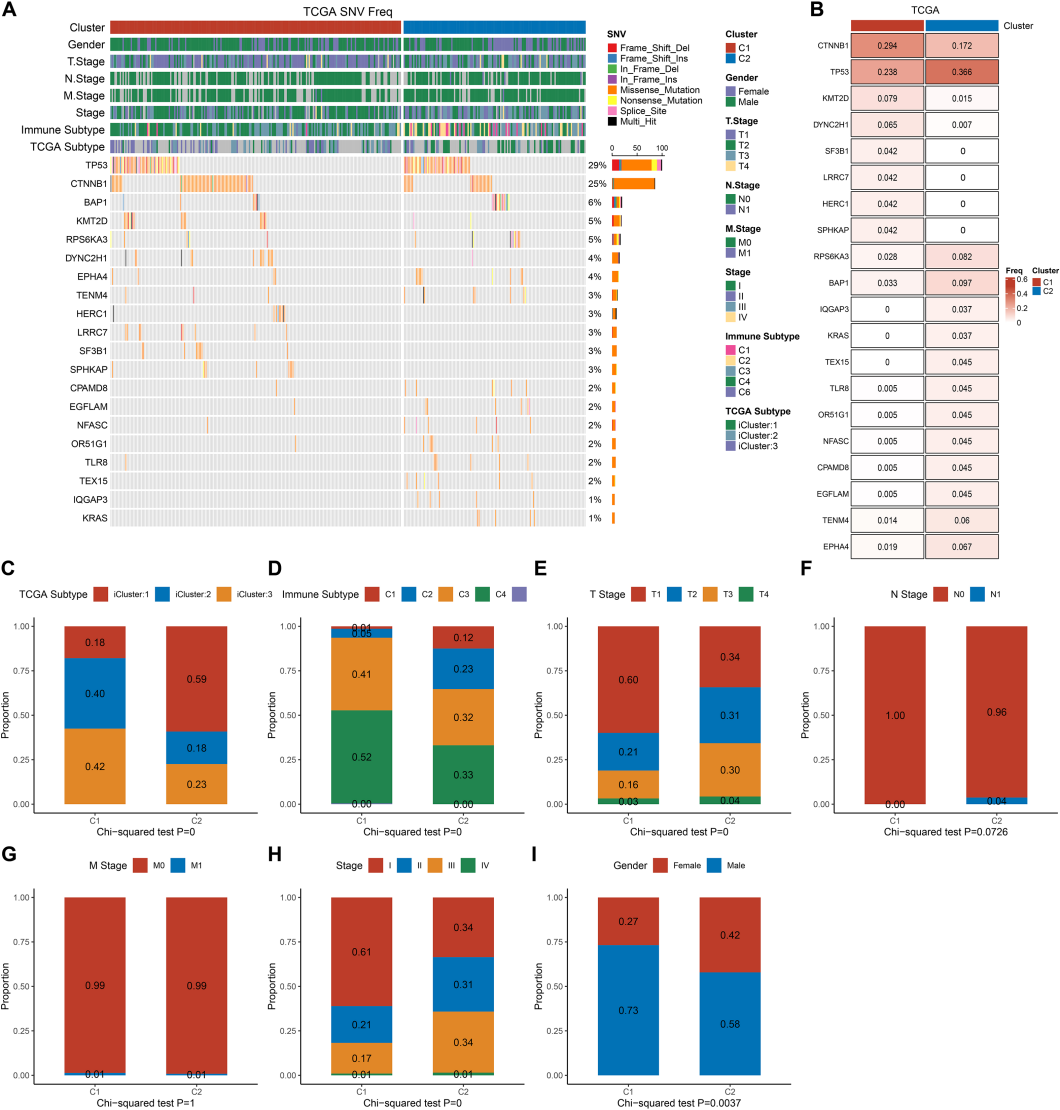
Genomic alterations and clinical associations of transcriptional subtypes in the TCGA cohort. **(A)** Oncoplot showing somatic mutation profiles of the top 25 frequently mutated genes across C1 and C2; **(B)** Comparison of mutation frequencies for individual genes between C1 and C2 subtypes; **(C)** Proportional distribution of TCGA molecular subtypes across C1 and C2 groups; **(D)** Proportional distribution of immune subtypes among C1 and C2 transcriptional subtypes; **(E)** Proportional distribution of T stage composition between C1 and C2; **(F)** Proportional distribution of N stage between C1 and C2 subtypes; **(G)** Proportional distribution of M stage between C1 and C2 subtypes; **(H)** Proportional distribution of overall clinical stage between C1 and C2 subtypes; **(I)** Gender distribution between C1 and C2 subtypes.

Somatic mutation analysis revealed that the C2 subtype exhibited significantly higher frequencies of TP53, BAP1, and RPS6KA3 mutations compared to the C1 subtype (**Figures 5A, B**). These mutations are known to be associated with aggressive tumor behavior and poor prognosis. Notably, TP53 mutation was markedly enriched in the C2 subtype (P < 0.001), suggesting a potential link between genomic instability and worse clinical outcome in this group.

Next, we assessed the distribution of immune subtypes across C1 and C2 clusters. The C2 subtype was significantly enriched in the TCGA iCluster3 subtype (**Figure 5C**). In terms of immune classification (26), C1 samples were predominantly composed of immune C2 (IFN-γ dominant) and C3 (inflammatory) subtypes, whereas C2 samples showed a higher proportion of immune C1 (wound healing) and C4 (lymphocyte-depleted) subtypes (**Figure 5D**, P< 0.001), indicating an immunosuppressive microenvironment in C2. The analysis of clinical parameters revealed that the C2 subtype was associated with more advanced tumor stages. Specifically, C2 samples exhibited a higher prevalence of T3/T4 tumors (P < 0.001, **Figure 5E**), an increased incidence of distant metastasis (M1, P = 0.009, **Figure 5G**), and a greater occurrence of stage III/IV cases (P < 0.001, **Figure 5H**). No significant difference was observed in N stage (lymph node involvement, P = 0.076, **Figure 5F**). Additionally, the gender distribution differed between subtypes, with male patients being more prevalent in the C2 subtype (P = 0.037, **Figure 5I**).

### Key gene screening and Mendelian randomization analysis

**Figure 6:**
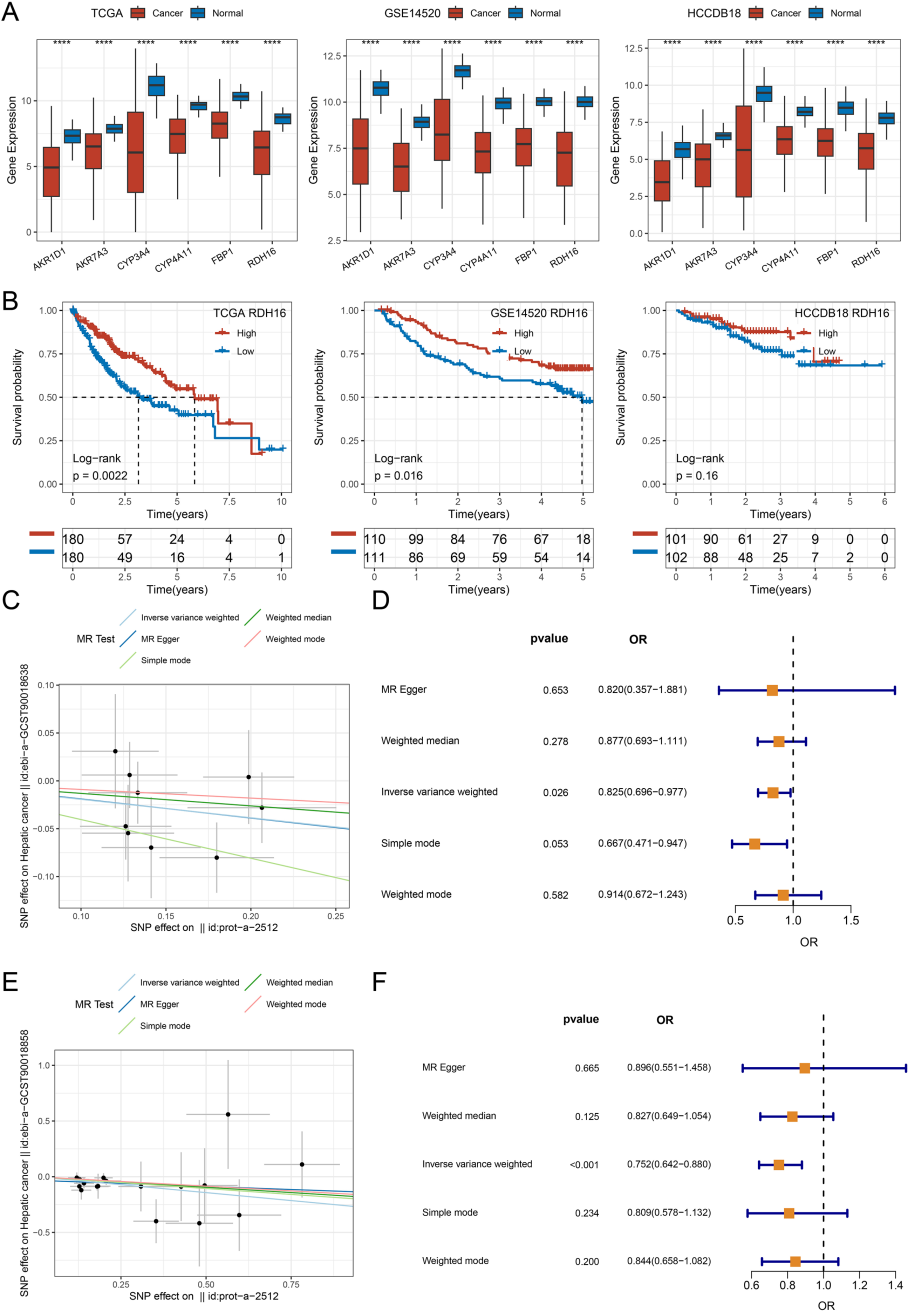
Prognostic significance and causal inference of RDH16 in HCC. **(A)** Expression of six genes in tumor versus normal liver tissues across multiple cohorts; **(B)** Kaplan-Meier survival analysis of HCC patients stratified by RDH16 expression in TCGA-LIHC, GSE14520, and HCCDB18 cohorts. Log-rank test was used to evaluate statistical significance; **(C)** A scatter plot illustrating the causal link between plasma RDH16 protein levels and HCC risk in the ebi-a-GCST90018638 dataset, as estimated by five Mendelian randomization (MR) methods; **(D)** Forest plot showing the odds ratios (ORs) and 95% confidence intervals for the link between RDH16 and HCC using various MR methods in the ebi-a-GCST90018638 dataset.; **(E)** Scatter plot showing the causal relationship between plasma RDH16 protein levels and HCC risk in the ebi-a-GCST9001885 dataset, estimated using five MR methods; **(F)** Forest plot presenting the ORs and 95% confidence intervals for the association between RDH16 and HCC across different MR methods in the ebi-a-GCST9001885 dataset. ****p <0.0001.

Univariate Cox analysis (p < 0.05) of the marker genes from Malignant-C3 across three datasets identified 39 genes (**Supplementary Figure 3**, **Supplementary Table 1**). We analyzed differentially expressed protein-coding genes between tumor and normal samples in the TCGA-LIHC, HCCDB18, and GSE14520 datasets to identify further key genes. Using a threshold of |log2FC| > 2 and FDR < 0.01, we identified six genes by intersecting these with the 39 previously identified genes. These six genes consistently showed low expression in tumor samples across all three datasets (**Figure 6A**). Kaplan-Meier survival analysis based on three independent HCC cohorts (TCGA, GSE14520, and HCCDB18) revealed that patients with high RDH16 expression had significantly better overall survival compared to those with low expression in the TCGA (p = 0.0022) and GSE14520 (p = 0.016) cohorts. A similar trend was observed in the HCCDB18 cohort (p = 0.16) (**Figure 6B**).

We conducted MR analysis using two independent datasets to assess the causal relationship between RDH16 and the risk of liver cancer. In Dataset 1(**Figures 6C, D**), the inverse variance weighted method revealed an odds ratio (OR) of 0.825 (95% CI: 0.696-0.977, P = 0.026), suggesting a protective effect of RDH16 against liver cancer. The Simple mode method showed a near-significant OR of 0.667 (95% CI: 0.471-0.947, P = 0.053), while the MR Egger and Weighted mode methods did not yield statistically significant results. In Dataset 2 (**Figures 6E, F**), the inverse variance weighted method again yielded a significant result (OR = 0.752, 95% CI: 0.642-0.880, P < 0.001), further supporting a protective role of RDH16 in liver cancer risk. The Weighted median method showed a consistent trend (OR = 0.827, 95% CI: 0.649-1.054, P = 0.125), although it was not statistically significant. Other methods, including MR Egger and Simple mode, did not show significance in this dataset.

In conclusion, using the inverse variance weighted method, both independent datasets demonstrated a significant inverse correlation between RDH16 and liver cancer risk. While results from other MR methods varied, the overall trend consistently indicated that RDH16 may exert a protective role in liver cancer development.

### Upregulated RDH16 predicts a favorable prognosis in hepatocellular carcinoma but is functionally dispensable *in vitro*

**Figure 7:**
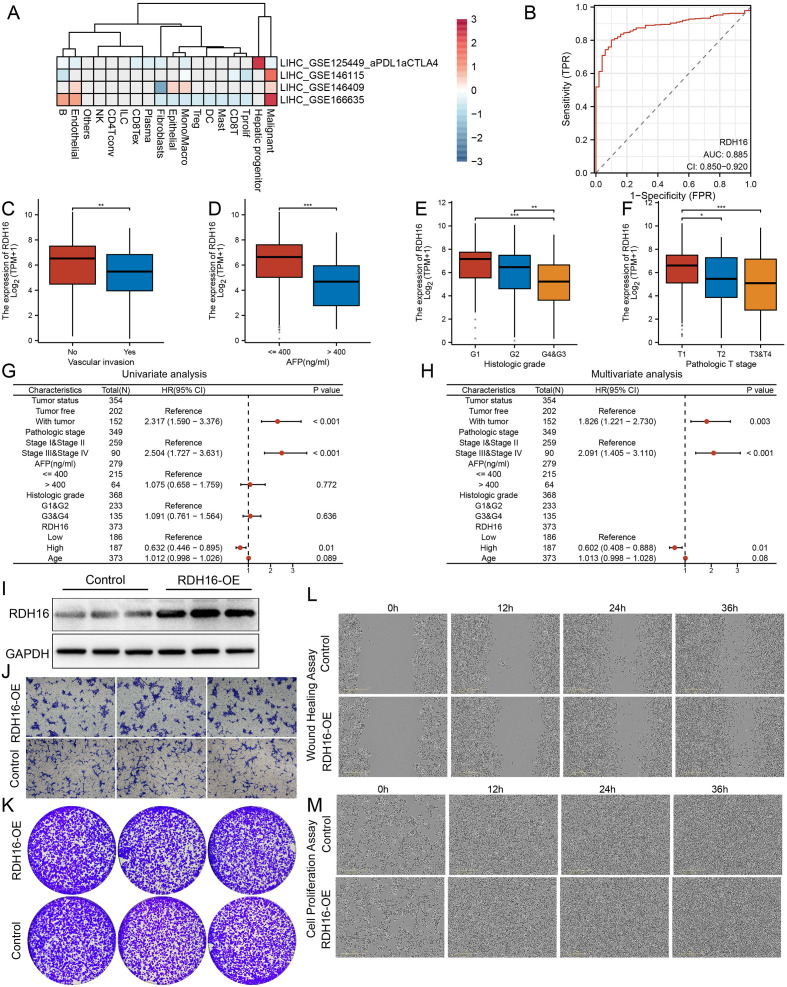
Clinical significance and functional validation of RDH16 in hepatocellular carcinoma. **(A)** Heatmap showing RDH16 expression across HCC datasets and immune subtypes, based on public cohort clustering; **(B)** ROC curve assessing the diagnostic efficacy of RDH16 in differentiating between tumor and normal tissues within the TCGA-LIHC cohort. (AUC = 0.885); C-F: Box plots comparing RDH16 expression levels with clinical features including vascular invasion **(C)**. serum AFP levels **(D)**. histologic grade **(E)**. and T stage **(F)**. in TCGA-LIHC cohort; **(G)** Uni-Cox regression analysis identifying clinical predictors of survival in HCC; **(H)** Multivariate Cox regression analysis confirming RDH16 as an independent prognostic indicator for overall survival; **(I)** Western blot validation of RDH16 protein overexpression in hepatoma cells after transfection; **(J)** Transwell invasion assay comparing invasive capacity between RDH16-overexpressing and control cells; **(K)** Colony formation assay assessing the clonogenic ability of RDH16-overexpressing versus control hepatoma cells; **(L)**. Wound healing assay monitoring cell migratory ability at 0, 12, 24, and 36 hours in RDH16-overexpressing and control cells; **(M)**. Cell proliferation assay evaluating growth kinetics over time in RDH16-overexpressing and control cells. *p< 0.05; **p<0.01; ***plt;0.001.

In three independent public single-cell datasets, we first analyzed the transcriptional level of RDH16. Clustering analysis revealed that RDH16 was primarily expressed in normal hepatocytes and malignant tumor cells within the tumor microenvironment (**Figure 7A**). ROC curve analysis based on the TCGA-LIHC cohort confirmed that RDH16 mRNA could robustly distinguish tumors from adjacent non-tumorous tissues (AUC = 0.885, 95% CI = 0.850-0.920) (**Figure 7B**). Further clinical correlation analyses demonstrated that low RDH16 expression was significantly associated with vascular invasion, AFP levels > 400 ng/ml, poor differentiation (grade G3/4), and advanced T stage (T3/4) (all P < 0.01) (**Figure 7C–F**). Cox regression analysis of 373 TCGA-LIHC patients identified high RDH16 expression as an independent favorable prognostic factor (multivariate HR = 0.602, 95% CI = 0.408-0.888, P = 0.01) (**Figures 7G, H**).

Furthermore, to validate the biological function of RDH16, we generated stable RDH16-overexpressing HCC cell lines. Western blot confirmed overexpression of RDH16 protein (**Figure 7I**); however, no significant differences between the RDH16-overexpressing and control groups were observed in transwell invasion, colony formation, wound healing, or proliferation assays (**Figures 7J–M**). These results suggest that modulating RDH16 expression alone *in vitro* is insufficient to alter the baseline migratory, invasive, or proliferative phenotypes of cancer cells.

### RDH16 exerts antitumor effects by modulating immune infiltration

**Figure 8:**
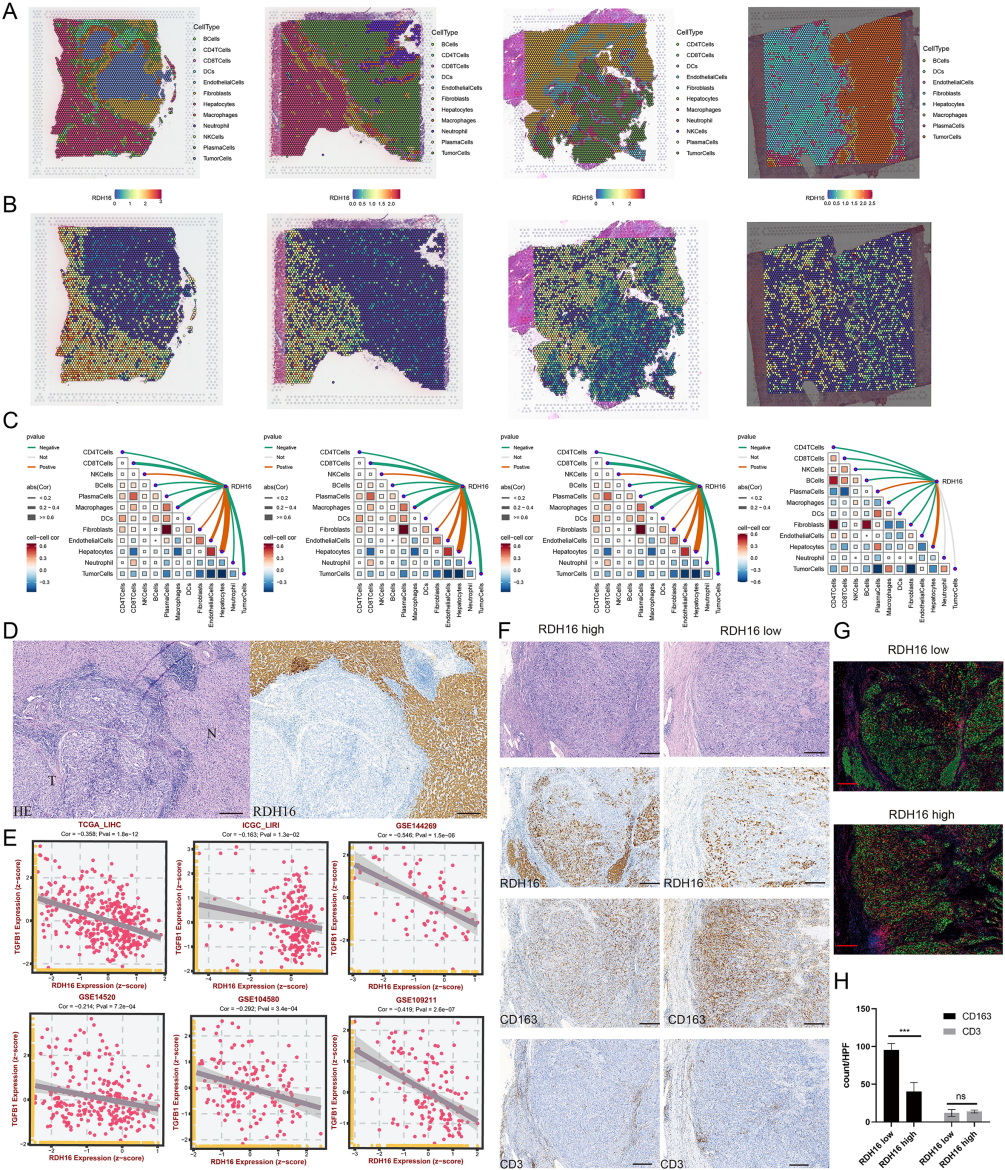
Spatial distribution and immune correlation of RDH16 expression in HCC. **(A)** Spatial transcriptomic data showing cell type annotation overlaid on tumor sections; **(B)** The expression and localization of RDH16 in spatial transcriptomic data; **(C)** The correlation between the expression level of the RDH16 gene and the content of different cell types based on spatial transcriptomic data; **(D)** Hematoxylin and eosin (HE) staining and Immunohistochemistry (IHC) staining for RDH16 in paired tumor (T) and adjacent normal (N) tissues; **(E)** Spearman correlation analysis between RDH16 and CD163 expression across multiple HCC cohorts, including TCGA_LIHC, ICGC_LIRI, and several GEO datasets; **(F)** Representative IHC images showing RDH16, CD163 (M2 macrophage marker), and CD3 (T cell marker) expression in RDH16-high versus RDH16-low tumors; **(G)** Multiplex immunofluorescence staining for CD163 (green) and CD3 (red) in RDH16-high and RDH16-low tumors to assess immune infiltration; **(H)** Quantification of CD163+ and CD3+ cell infiltration from multiplex immunofluorescence data. An unpaired t-test assessed statistical significance. ns, p≥0.05; ***p<0.001.

Spatial transcriptomic analysis of four HCC samples revealed distinct cell type compositions across different tissue microenvironments, delineating the cellular architecture of the tumor at high spatial resolution (**Figure 8A**). 11 cell types were identified and mapped across the tissue sections. Notably, RDH16 exhibited marked spatial heterogeneity, with high expression regions largely colocalized with hepatocyte-enriched zones. In contrast, tumor regions showed relatively low levels of RDH16 expression (**Figure 8B**). Further analysis revealed a significant positive correlation between RDH16 expression and the proportion of hepatocytes within each spatial spot, which is highly consistent with the previous cell localization analysis. Moreover, RDH16 expression was significantly negatively correlated with the infiltration levels of various immune cell types within spatial spots. These findings suggest that RDH16 may be essential in regulating immune infiltration within the tumor microenvironment (**Figure 8C**).

Immunohistochemical analysis of normal (N) and tumor (T) liver tissues shows that RDH16 exhibits brownish-yellow granular cytoplasmic staining in normal hepatocytes, with expression levels significantly higher than in HCC tissues (**Figure 8D**). In six independent HCC cohorts (TCGA-LIHC, ICGC-LIRI, GSE144269, GSE14520, GSE109211and GSE104580), we performed correlation analyses between the expression levels of RDH16 and TGFB1. Consistently, RDH16 expression was significantly negatively correlated with TGFB1 expression across all cohorts, with correlation coefficients ranging from -0.16 to -0.55 (all p-values < 0.05). These results suggest that elevated RDH16 expression is commonly associated with reduced TGFB1 levels, implying that RDH16 upregulation may suppress immune evasion in the HCC tumor microenvironment by alleviating TGFB1-mediated immunosuppression (**Figure 8E**). Immunohistochemistry shows intra-tumoral expression of CD163 and CD3 in RDH16-high versus RDH16-low groups (CD163 marks M2 macrophages; CD3 marks T cells) (**Figure 8F**). Compared with RDH16-high regions, RDH16-low regions exhibit a significant increase in CD163^+^ macrophages, whereas CD3^+^ T-cell unchanged. The corresponding multiplex immunofluorescence results for CD163 and CD3 in RDH16-high and RDH16-low tumor regions are presented (**Figure 8G**). The bar graph reveals a significant increase (p<0.0011) in CD163-positive cells in low versus high RDH16 expression areas, whereas CD3 levels remain unchanged (**Figure 8H**), which suggests a link between reduced RDH16 and M2 macrophage infiltration, potentially contributing to local immunosuppression in HCC.

## Discussion

Single-cell transcriptomic analysis of HCC reveals key insights into tumor heterogeneity and the immune microenvironment. The study precisely identifies nine major cell types, including B cells, T cells, NK cells, macrophages, and fibroblasts. Malignant cells, macrophages, and fibroblasts are significantly enriched in tumor tissues, whereas non-tumor regions are dominated by B and T cells. Critically, overexpression of RDH16 does not directly affect tumor cell proliferation, migration, or invasion. However, high RDH16 expression in normal liver tissue correlates with CD3+ lymphocyte infiltration, while its absence in tumor regions negatively correlates with CD163+ histiocytic infiltration. Rather than the lack of T lymphocyte expression, it is hypothesized that RDH16 overexpression enhances antitumor immunity mediated by T lymphocytes. In contrast, the absence of RDH16 may promote M2 macrophage infiltration, which is detrimental to the tumor immune response.

This study’s key innovation lies in the systematic application of single-cell transcriptomics to HCC research, comprehensively elucidating tumor heterogeneity and the complexity of the immune microenvironment. It reveals the functional characteristics of different cell types (e.g., tumor and immune cells) and their impact on tumor progression, investigates dynamic changes in immune cell signaling pathways across various stages of HCC, and identifies prognostic biomarkers. Furthermore, when studying the immune microenvironment of HCC, single-cell RNA sequencing reveals the mechanism by which tumor-associated macrophages inhibit T cell infiltration and identifies critical molecular interactions that regulate immunosuppression, offering potential targets for immunotherapy. Additionally, single-cell transcriptomics reveals intra- and inter-tumoral heterogeneity in HCC, particularly within immunosuppressive microenvironments.

Using single-cell analysis, this study uncovers a novel role for RDH16 in regulating tumor immune infiltration, particularly in promoting M2 macrophage and suppressive T cell infiltration. Firstly, RDH16 expression is significantly downregulated in hepatocellular carcinoma, correlating with poor patient prognosis, suggesting a tumor-suppressive function. Research indicates that RDH16 inhibits tumor cell growth and migration by increasing retinoic acid levels and blocking fatty acid synthesis (15), which is potentially linked to M2 macrophage polarization, which typically exhibits pro-tumor properties in the tumor microenvironment (27). However, the mechanism by which RDH16 regulates T cell infiltration remains unclear.

This study elucidates the complex cellular composition and immune dynamics in HCC, providing a theoretical basis for personalized treatment strategies, such as those tailored to patients’ specific immune microenvironments. To understand the basis for these customized treatment strategies, exploring the nature of the tumor immune microenvironment in HCC is essential. The tumor immune microenvironment in HCC is not a static backdrop but a highly complex and heterogeneous system. Research demonstrates significant spatial heterogeneity within HCC’s immune microenvironment, encompassing immune cell infiltration patterns and functional states, which directly influence tumor immune evasion and are closely associated with treatment resistance (28). By deeply analyzing HCC’s immune microenvironment and identifying key immune cell subsets and their functional states, more precise therapeutic strategies can be developed, thereby enhancing patient outcomes and survival rates (29).

RDH16, a member of the retinol dehydrogenase family, is integral to RA metabolism, positioning it at a metabolic-immune interface in HCC. RA signaling is a key regulator of the tumor immune microenvironment, influencing the polarization of tumor-associated macrophages and the activation of T cells. Consistent with this, all−trans retinoic acid has been reported to limit TAM polarization toward the immunosuppressive M2 phenotype, supporting the notion that disruptions in RA metabolism can remodel the immune landscape of HCC (30). In our study, we observed that HCC tissues with diminished expression of RDH16 exhibited a marked increase in the infiltration of immunosuppressive CD163^+^ M2 macrophages, alongside a reduction in CD3^+^ T cell infiltration. These findings suggest that the downregulation of RDH16 may inhibit RA signaling, thereby promoting an immunosuppressive tumor microenvironment and facilitating immune evasion. Collectively, the metabolic abnormalities arising from RDH16 downregulation may constitute a pivotal mechanism through which metabolic reprogramming influences the immune axis in HCC. This mechanism is analogous to other metabolic-immune interactions, such as those mediated by IGF2BP1 and PRMT3 (31), highlighting the essential role of metabolic reprogramming in immune escape during the progression of HCC.

In HCC treatment, RDH16’s pivotal role in regulating macrophage and T-cell infiltration offers a crucial direction for developing new targeted intervention strategies. Research indicates that RDH16, a tumor suppressor gene, is significantly downregulated in HCC, correlating with a poor patient prognosis (15). Macrophage and T-cell infiltration are key factors in HCC immunotherapy. Studies show that modulating macrophage polarization can substantially impact T-cell infiltration and function (32, 33). For instance, targeting the CCL2/CCR2 signaling pathway can reprogram tumor-associated macrophages (TAMs), thereby enhancing immune cell infiltration and antitumor immune responses (34). Thus, activating RDH16 may improve the HCC immune microenvironment, boosting immune cell infiltration and antitumor activity. RDH16 agonists or combined immunotherapies hold promise for overcoming the current limitations of HCC treatment.

Although this study highlights the critical role of RDH16 in HCC, several negative findings warrant attention. First, in the HCCDB18 cohort, the association between RDH16 expression and patient survival did not reach statistical significance (p = 0.16). We speculate that this result may be attributed to the intrinsic heterogeneity of the cohort: HCCDB18 includes patients with diverse etiological backgrounds, clinical stages, and treatment regimens. Its relatively short follow-up period and limited sample size may reduce statistical power, thereby masking the true prognostic trend. Nonetheless, the majority of independent cohorts consistently support the prognostic relevance of RDH16. Second, our Mendelian randomization analysis using the inverse variance weighted method suggested a potential protective effect of higher RDH16 expression against HCC. In contrast, results from specific approaches, such as the MR-Egger method, did not achieve statistical significance. It is important to note that such discrepancies among MR methods are not uncommon, as they rely on different underlying assumptions. The IVW approach offers greater statistical efficiency but is more susceptible to horizontal pleiotropy. In contrast, MR-Egger can test for pleiotropy but often yields less precise estimates with wider confidence intervals. Furthermore, factors such as weak instrumental variable strength, differences in linkage disequilibrium structure, and potential heterogeneity among GWAS cohorts may also contribute to the observed inconsistencies. Therefore, while the overall MR evidence supports a possible protective role of RDH16 in HCC, this causal relationship should be further validated in larger, more homogeneous cohorts using stronger and more reliable instrumental variables.

Moreover, this study has several limitations. First, despite integrating multiple public resources (TCGA, GEO) and single-cell datasets, the overall sample size—particularly for the spatial transcriptomics and immunohistochemistry analyses—remains limited, which may restrict the generalizability of our conclusions. Therefore, validation in larger, independent cohorts is warranted. Second, single−cell transcriptomic analyses are highly sensitive to data quality, and technical variables (e.g., tissue processing, sequencing depth, and batch effects) can introduce bias; moreover, although the immunomodulatory role of RDH16 was supported by clinical specimens and *in vitro* assays, *in vivo* confirmation in appropriate animal models is still required. Third, our IHC profiling primarily focused on CD163^+^ M2 macrophages and pan−CD3^+^ T cells; we did not fully delineate the distribution of CD8^+^ T cells, regulatory T cells, dendritic cells, or natural killer cells in RDH16−high versus RDH16−low tumors. In future work, we plan to employ a broader marker panel to refine immune-cell annotation and integrate these data with tumor mutational burden, microsatellite instability status, and immune-checkpoint gene expression to construct a more comprehensive RDH16−associated immune landscape.

In addition, given the close association between RDH16 and the remodeling of the tumor immune microenvironment, RDH16 may influence the efficacy of immune checkpoint inhibitor therapy. In this study, tumors with high RDH16 expression exhibited reduced infiltration of immunosuppressive macrophages and enhanced T-cell activity, suggesting that RDH16 may promote an activated immune state. Therefore, we cautiously believe that therapeutic strategies that enhance the function or expression of RDH16 may work in synergy with PD-1/PD-L1 inhibitors, thereby increasing the responsiveness of immunotherapy. Although no clinical drugs currently target RDH16 directly, interventions that modulate retinoic acid metabolism or reprogram macrophages may offer feasible approaches. It is also highly anticipated to evaluate the therapeutic potential of combining RDH16-based regulation with immune checkpoint blockade in additional animal models. Furthermore, several studies have highlighted the limitations of conventional diagnostic approaches. Integrating RDH16 with established clinical indicators may offer enhanced diagnostic performance and improved clinical outcomes (35). Most critically, the exact molecular pathways by which RDH16 influences macrophage and T-cell infiltration, such as through retinoic acid metabolism or cytokine networks, remain unclear. These limitations could impact how broadly the results can be applied and the strength of the mechanistic conclusions drawn from the study. Therefore, it is essential to conduct further investigations, including multicenter cohort validations and targeted functional experiments, to enhance the reliability and applicability of the findings.

## Conclusion

Using single-cell transcriptomics, this article systematically analyzes the heterogeneity and immune microenvironment of HCC, revealing a novel mechanism of RDH16 in regulating immune infiltration. These findings deepen our understanding of HCC pathogenesis and provide new directions and potential therapeutic targets for future clinical treatments. Despite some limitations, such as the small sample size and limited follow-up time, the study’s innovation and clinical significance offer crucial insights for HCC research and therapy.

## Data Availability

The original contributions presented in the study are included in the article/**Supplementary Material**. Further inquiries can be directed to the corresponding author.

## References

[1] Singh SP Madke T Chand P . Global epidemiology of hepatocellular carcinoma. J Clin Exp Hepatology. (2025) 15:102446. doi: 10.1016/j.jceh.2024.102446, PMID: 39659901 PMC11626783

[2] Chan L-K Tsui Y-M Ho DW-H Ng IO-L . Cellular heterogeneity and plasticity in liver cancer. Semin Cancer Biol. (2022) 82:134–49. doi: 10.1016/j.semcancer.2021.02.015, PMID: 33647386

[3] Flavahan WA Gaskell E Bernstein BE . Epigenetic plasticity and the hallmarks of cancer. Science. (2017) 357:eaal2380. doi: 10.1126/science.aal2380, PMID: 28729483 PMC5940341

[4] Budhu A Pehrsson EC He A Goyal L Kelley RK Dang H . Tumor biology and immune infiltration define primary liver cancer subsets linked to overall survival after immunotherapy. Cell Rep Med. (2023) 4:101052. doi: 10.1016/j.xcrm.2023.101052, PMID: 37224815 PMC10313915

[5] Varanasi SK Chen D Liu Y Johnson MA Miller CM Ganguly S . Bile acid synthesis impedes tumor-specific T cell responses during liver cancer. Science. (2025) 387:192–201. doi: 10.1126/science.adl4100, PMID: 39787217 PMC12166762

[6] Huang A Lv B Zhang Y Yang J Li J Li C . Construction of a tumor immune infiltration macrophage signature for predicting prognosis and immunotherapy response in liver cancer. Front Mol Biosci. (2022) 9. doi: 10.3389/fmolb.2022.983840, PMID: 36120553 PMC9479109

[7] Zheng H Peng X Yang S Li X Huang M Wei S . Targeting tumor-associated macrophages in hepatocellular carcinoma: biology, strategy, and immunotherapy. Cell Death Discovery. (2023) 9:65. doi: 10.1038/s41420-023-01356-7, PMID: 36792608 PMC9931715

[8] Gong Q Chen X Liu F Cao Y . Machine learning-based integration develops a neutrophil-derived signature for improving outcomes in hepatocellular carcinoma. Front Immunol. (2023) 14. doi: 10.3389/fimmu.2023.1216585, PMID: 37575244 PMC10419218

[9] Zhang P Wang L Liu H Lin S Guo D . Unveiling the crucial role of glycosylation modification in lung adenocarcinoma metastasis through artificial neural network-based spatial multi-omics single-cell analysis and Mendelian randomization. BMC Cancer. (2025) 25:249. doi: 10.1186/s12885-025-13650-x, PMID: 39948531 PMC11823056

[10] Ji D Lu S Zhang H Li Z Wang S Miao T . Bulk and single-cell transcriptome reveal the immuno-prognostic subtypes and tumour microenvironment heterogeneity in HCC. Liver Int. (2024) 44:979–95. doi: 10.1111/liv.15828, PMID: 38293784

[11] Jiang G Tu J Zhou L Dong M Fan J Chang Z . Single-cell transcriptomics reveal the heterogeneity and dynamic of cancer stem-like cells during breast tumor progression. Cell Death Disease. (2021) 12:979. doi: 10.1038/s41419-021-04261-y, PMID: 34675206 PMC8531288

[12] Ding P Li L Li L Lv X Zhou D Wang Q . C5aR1 is a master regulator in Colorectal Tumorigenesis via Immune modulation. Theranostics. (2020) 10:8619–32. doi: 10.7150/thno.45058, PMID: 32754267 PMC7392014

[13] Jiang L Jiang Y Zhou X Wang L Zhang S Jiang C . The key role of COA6 in pancreatic ductal adenocarcinoma: metabolic reprogramming and regulation of the immune microenvironment. J Cell Mol Med. (2025) 29:e70685. doi: 10.1111/jcmm.70685, PMID: 40596639 PMC12213452

[14] Zhang P Wang L Lin H Han Y Zhou J Song H . Integrative multiomics analysis reveals the subtypes and key mechanisms of platinum resistance in gastric cancer: identification of KLF9 as a promising therapeutic target. J Trans Med. (2025) 23:877. doi: 10.1186/s12967-025-06725-7, PMID: 40775648 PMC12330134

[15] Zhu Y-H Li J-B Wu R-Y Yu Y Li X Li Z-L . Clinical significance and function of RDH16 as a tumor-suppressing gene in hepatocellular carcinoma. Hepatol Res. (2020) 50:110–20. doi: 10.1111/hepr.13432, PMID: 31661588

[16] Zhang HZ Hao SL Yang WX . How does retinoic acid (RA) signaling pathway regulate spermatogenesis? Histol Histopathol. (2022) 37:1053–64. doi: 10.14670/HH-18-478, PMID: 35673893

[17] Devalaraja S To TKJ Folkert IW Natesan R Alam MZ Li M . Tumor-derived retinoic acid regulates intratumoral monocyte differentiation to promote immune suppression. Cell. (2020) 180:1098–114.e16. doi: 10.1016/j.cell.2020.02.042, PMID: 32169218 PMC7194250

[18] Schlecker E Stojanovic A Eisen C Quack C Falk CS Umansky V . Tumor-infiltrating monocytic myeloid-derived suppressor cells mediate CCR5-dependent recruitment of regulatory T cells favoring tumor growth. J Immunol. (2012) 189:5602–11. doi: 10.4049/jimmunol.1201018, PMID: 23152559

[19] Liu L Huo S Liu J Li Q Wang J . Metabolic reprogramming of myeloid-derived suppressor cells in the tumor microenvironment. Discov Med. (2021) 31:141–6. 35188888

[20] Jiang Z Wu Y Miao Y Deng K Yang F Xu S . HCCDB v2.0: decompose expression variations by single-cell RNA-seq and spatial transcriptomics in HCC. Genomics Proteomics Bioinf. (2024) 22:qzae011. doi: 10.1093/gpbjnl/qzae011, PMID: 38886186 PMC11423853

[21] Roessler S Long EL Budhu A Chen Y Zhao X Ji J . Integrative genomic identification of genes on 8p associated with hepatocellular carcinoma progression and patient survival. Gastroenterology. (2012) 142:957–66.e12. doi: 10.1053/j.gastro.2011.12.039, PMID: 22202459 PMC3321110

[22] Liu Z Liu L Weng S Xu H Xing Z Ren Y . BEST: a web application for comprehensive biomarker exploration on large-scale data in solid tumors. J Big Data. (2023) 10:165. doi: 10.1186/s40537-023-00844-y

[23] Lu Y Yang A Quan C Pan Y Zhang H Li Y . A single-cell atlas of the multicellular ecosystem of primary and metastatic hepatocellular carcinoma. Nat Commun. (2022) 13:4594. doi: 10.1038/s41467-022-32283-3, PMID: 35933472 PMC9357016

[24] He X Liu F Gong Q . Identification of a senescence-related transcriptional signature to uncover molecular subtypes and key genes in hepatocellular carcinoma. PloS One. (2024) 19:e0311696. doi: 10.1371/journal.pone.0311696, PMID: 39383169 PMC11463828

[25] Gu Z . Complex heatmap visualization. iMeta. (2022) 1:e43. doi: 10.1002/imt2.43, PMID: 38868715 PMC10989952

[26] Thorsson V Gibbs DL Brown SD Wolf D Bortone DS Ou Yang T-H . The immune landscape of cancer. Immunity. (2018) 48:812–30.e14. doi: 10.1016/j.immuni.2018.03.023, PMID: 29628290 PMC5982584

[27] Chen Y Qi Y Jiang Y Li Y Yang S Wang L . Icariin modulates the tumor microenvironment in colorectal cancer by targeting M2 macrophage polarization via PI3K/AKT pathway. Bioorganic Medicinal Chem. (2025) 129:118317. doi: 10.1016/j.bmc.2025.118317, PMID: 40683071

[28] Bai Y Chen D Cheng C Li Z Chi H Zhang Y . Immunosuppressive landscape in hepatocellular carcinoma revealed by single-cell sequencing. Front Immunol. (2022) 13. doi: 10.3389/fimmu.2022.950536, PMID: 35967424 PMC9365996

[29] Song W Li M Liu W Xu W Zhou H Wei S . Role of immune cell homeostasis in research and treatment response in hepatocellular carcinoma. Clin Exp Med. (2025) 25:42. doi: 10.1007/s10238-024-01543-5, PMID: 39826024 PMC11742861

[30] Shao XJ Xiang SF Chen YQ Zhang N Cao J Zhu H . Inhibition of M2-like macrophages by all-trans retinoic acid prevents cancer initiation and stemness in osteosarcoma cells. Acta Pharmacol Sin. (2019) 40:1343–50. doi: 10.1038/s41401-019-0262-4, PMID: 31296953 PMC6786412

[31] Ding CH Yan FZ Xu BN Qian H Hong XL Liu SQ . PRMT3 drives PD-L1-mediated immune escape through activating PDHK1-regulated glycolysis in hepatocellular carcinoma. Cell Death Dis. (2025) 16:158. doi: 10.1038/s41419-025-07482-7, PMID: 40050608 PMC11885674

[32] DeNardo DG Ruffell B . Macrophages as regulators of tumour immunity and immunotherapy. Nat Rev Immunol. (2019) 19:369–82. doi: 10.1038/s41577-019-0127-6, PMID: 30718830 PMC7339861

[33] Savage P . Macrophage modulation of tumor immunity. Science. (2024) 386:850–1. doi: 10.1126/science.adt5661, PMID: 39571039

[34] Avila MA Berasain C . Targeting CCL2/CCR2 in tumor-infiltrating macrophages: A tool emerging out of the box against hepatocellular carcinoma. Cell Mol Gastroenterol Hepatology. (2019) 7:293–4. doi: 10.1016/j.jcmgh.2018.11.002, PMID: 30529279 PMC6354282

[35] Chen Y Yan H Xu Y Chen K Yang R Yang J . Analysis of the predictive value of the prostate-specific antigen-to-neutrophil ratio for the diagnosis of prostate cancer. Discover Oncol. (2025) 16:13. doi: 10.1007/s12672-025-01760-8, PMID: 39762493 PMC11704101

