# Supplementary figures and images for: Single-cell transcriptomics reveals a novel mechanism of RDH16 regulating immune infiltration in hepatocellular carcinoma
Source: Front Immunol. 2026 Jan 12;16:1689987. doi: 10.3389/fimmu.2025.1689987 (PMC12832884; doi:10.3389/fimmu.2025.1689987)

A

nFeature\_RNA

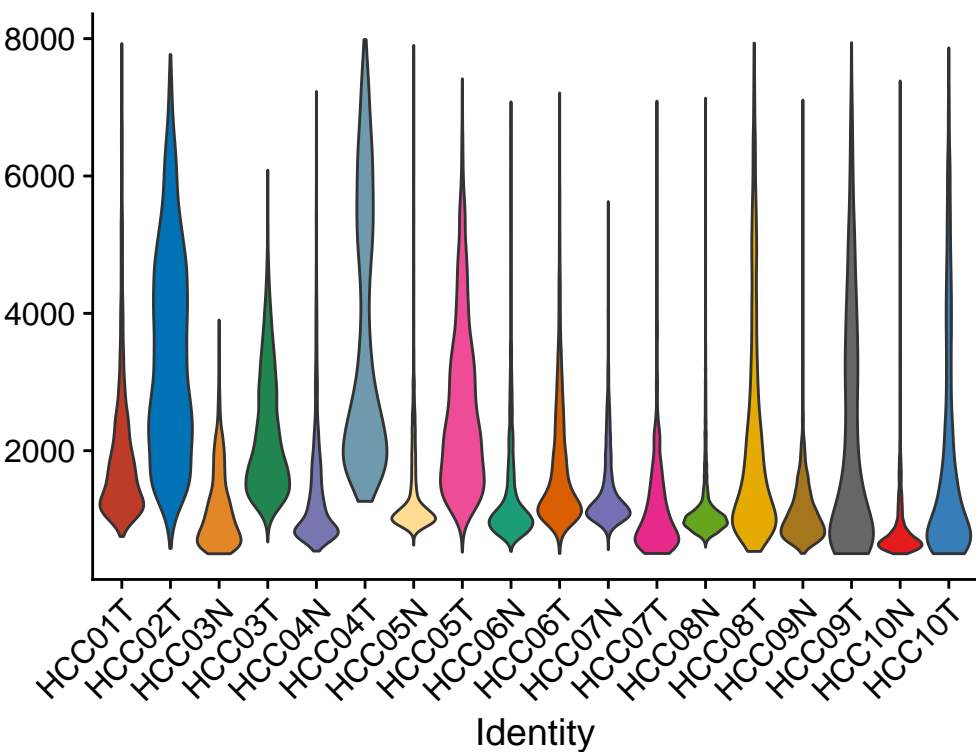

nCount\_RNA

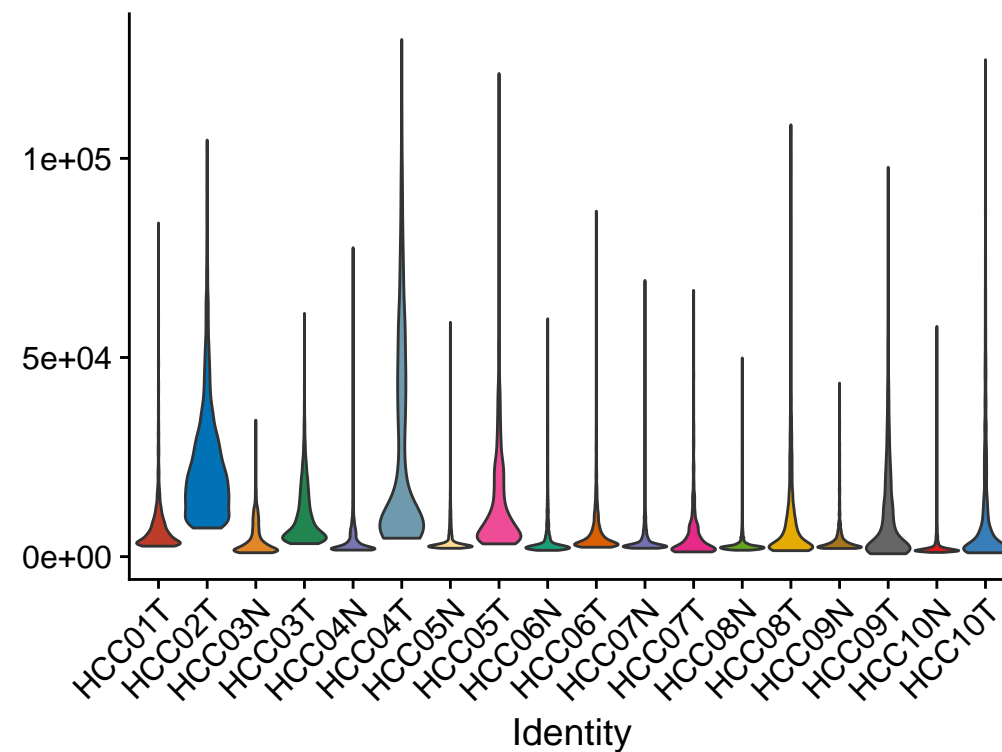

percent.mt

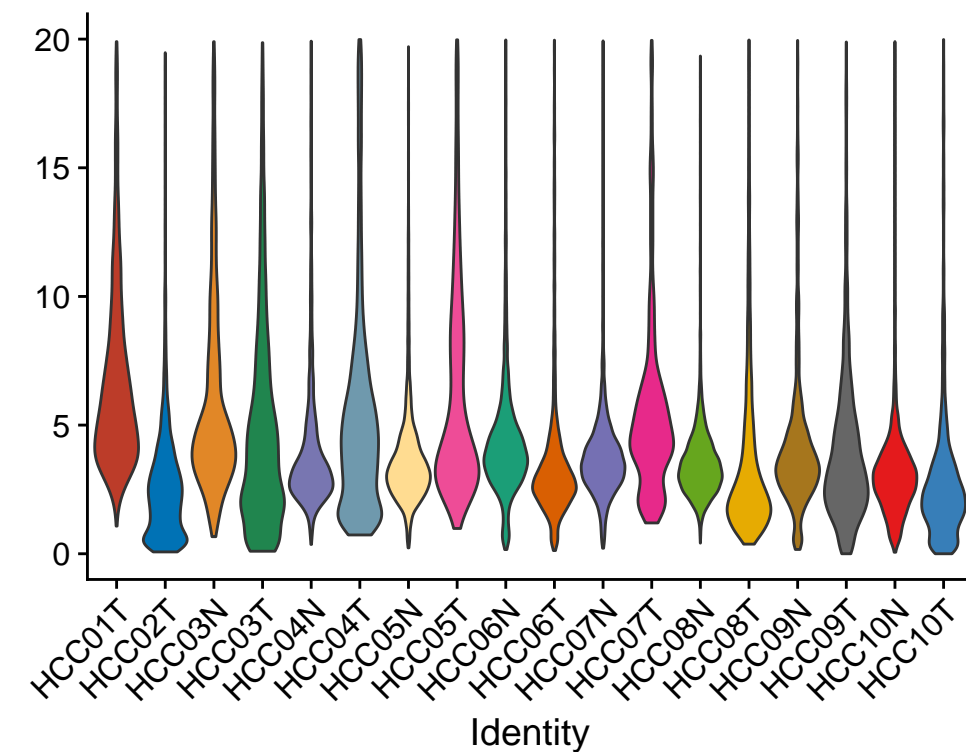

B

nFeature\_RNA

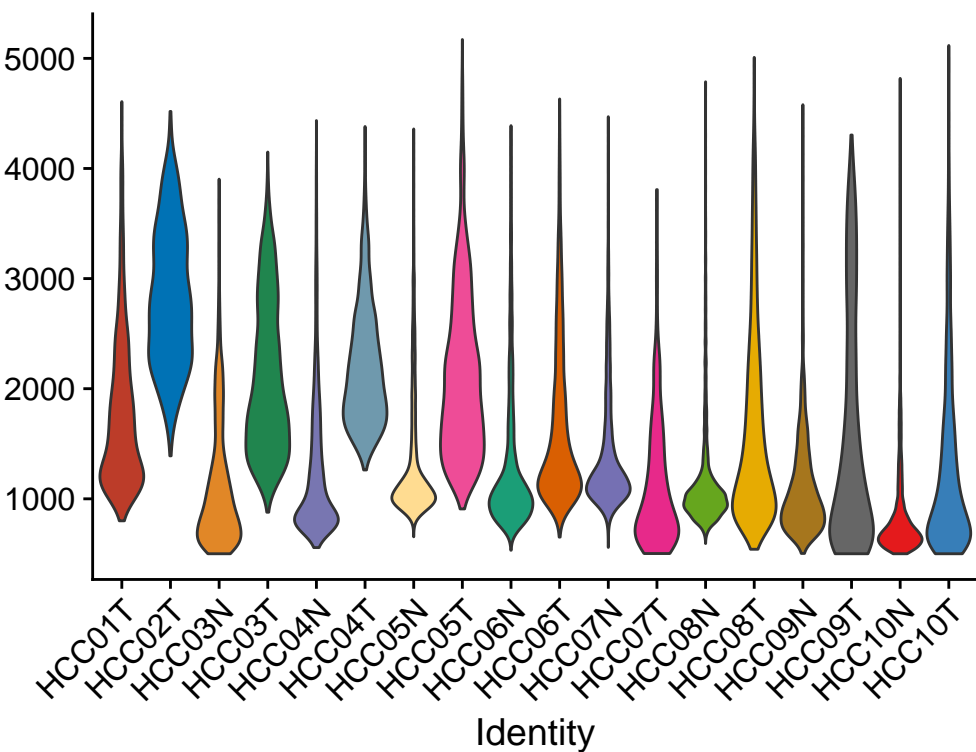

nCount\_RNA

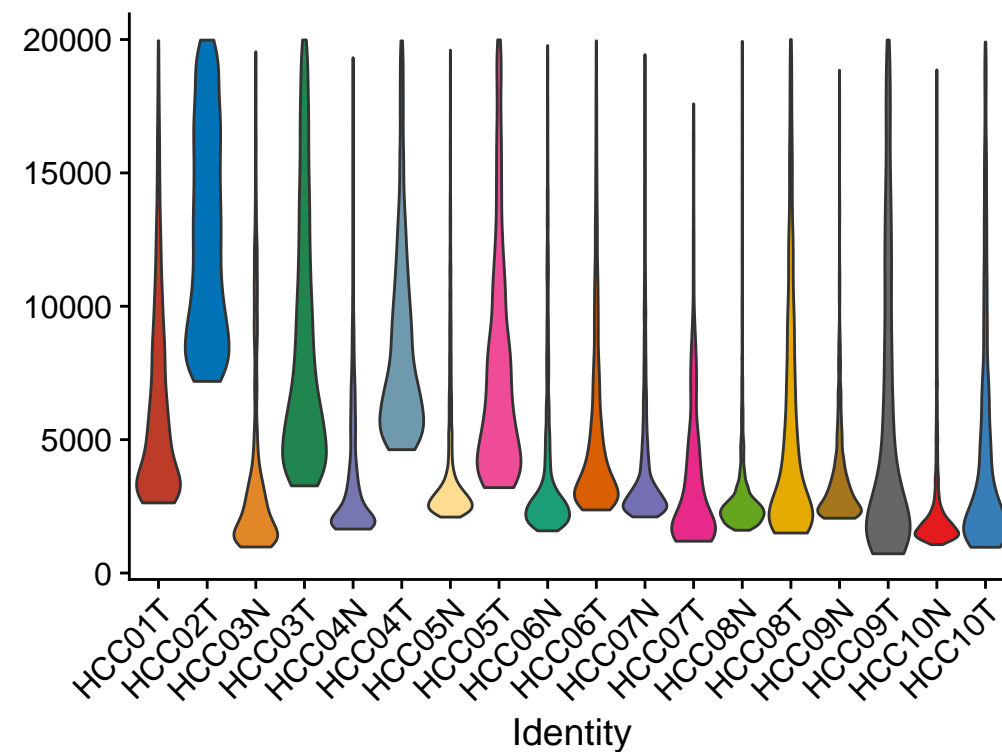

percent.mt

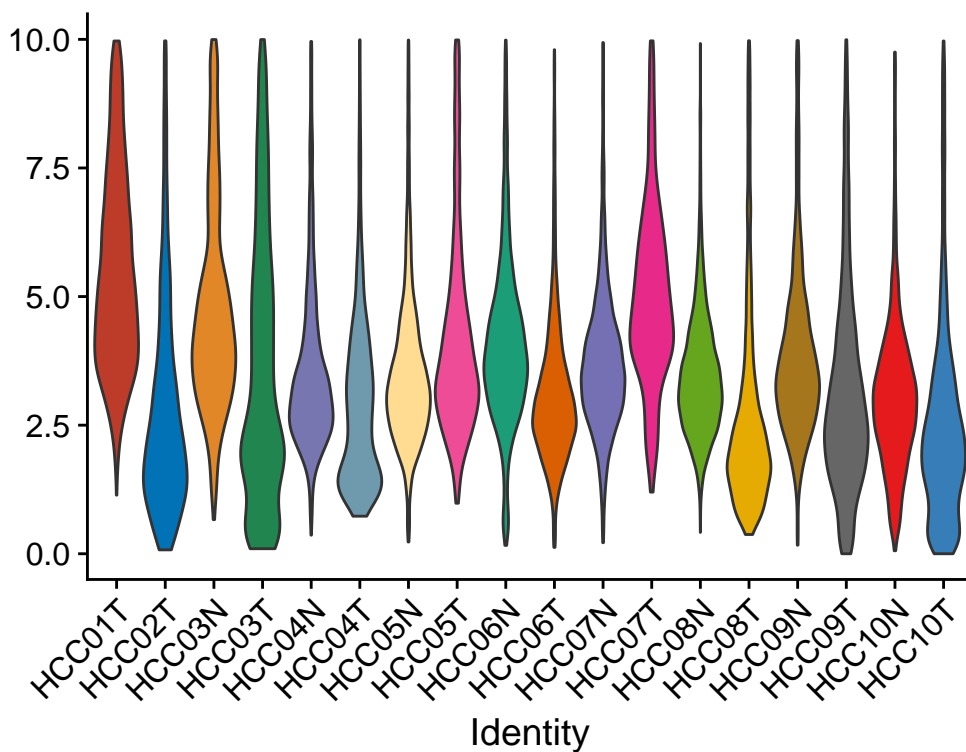

Supplement: Supplementary file 1 [file DataSheet1.pdf]

Stat raw clean

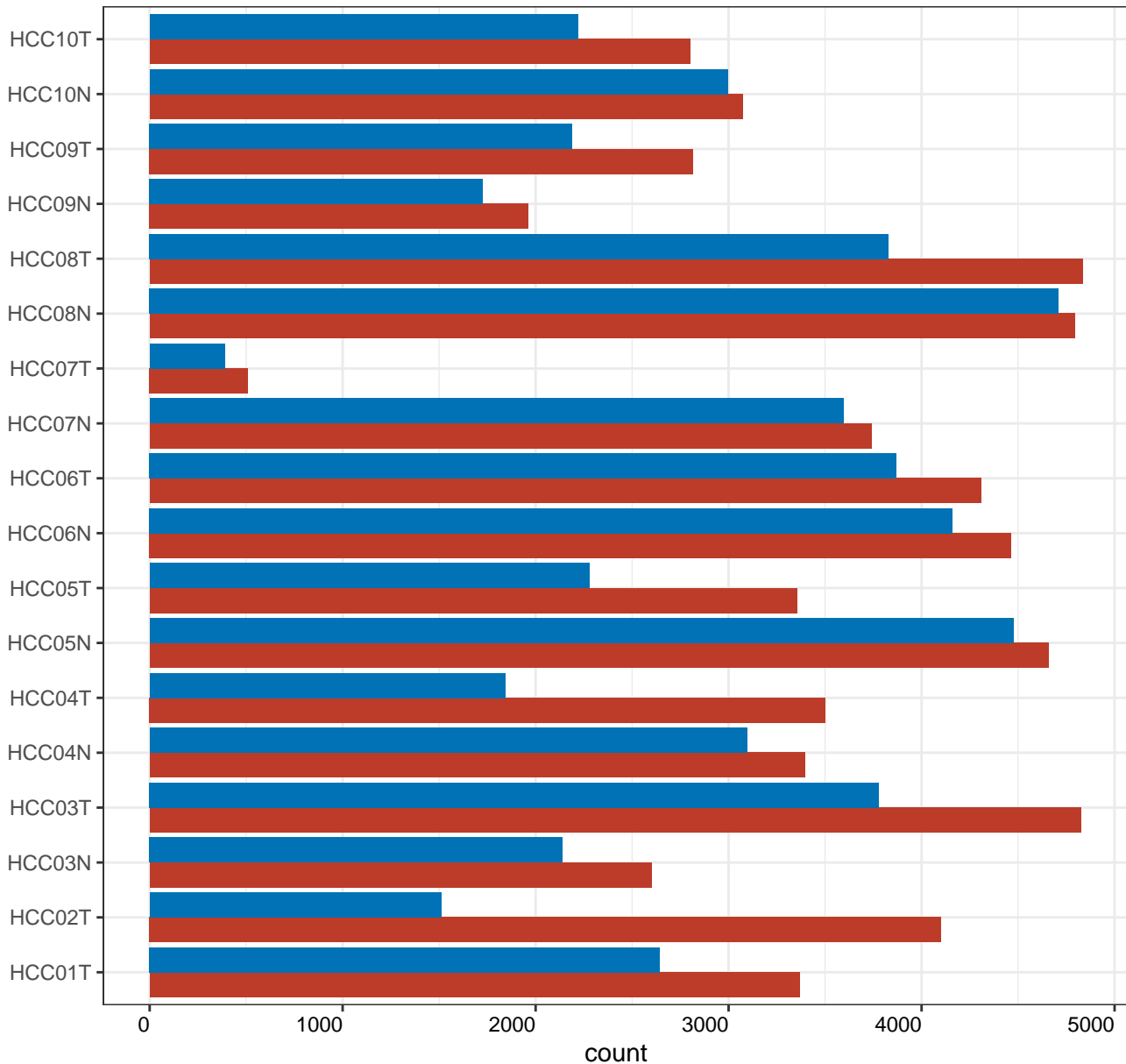

Supplement: Supplementary file 2 [file DataSheet2.pdf]

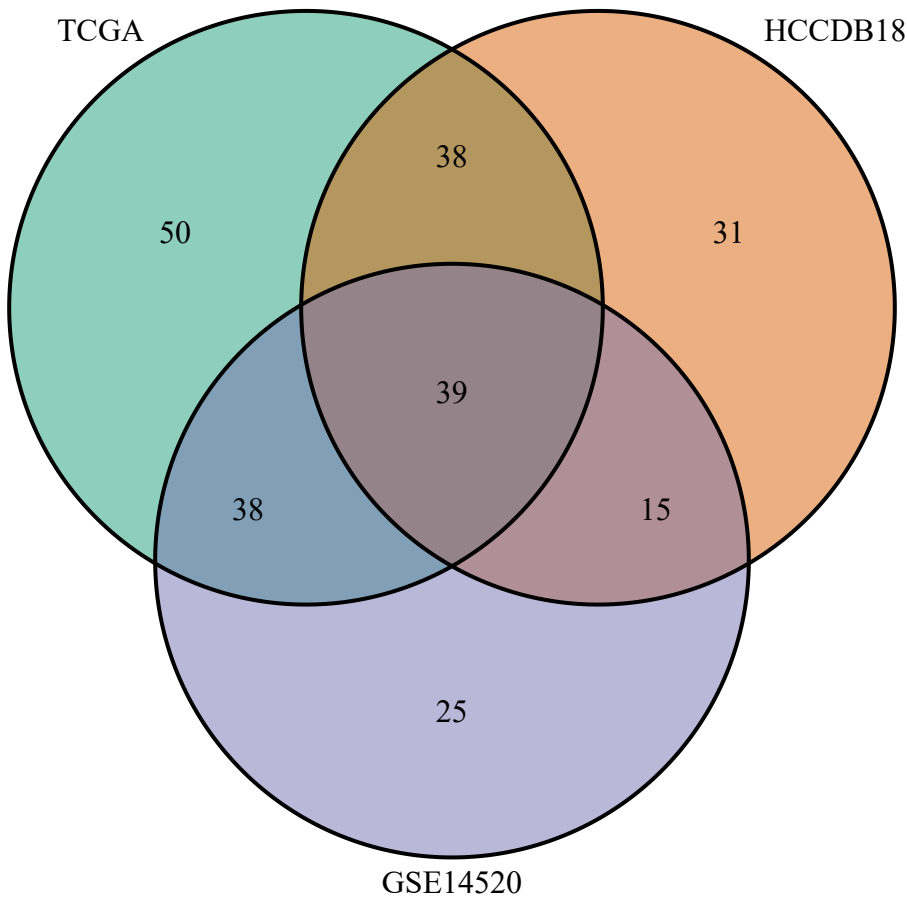

Supplement: Supplementary file 3 [file DataSheet3.pdf]
